# Social, Geographical and Income Inequality as Demonstrated by the Coronary Calcium Score: An Ecological Study in Sydney, Australia

**DOI:** 10.3390/ijerph20095699

**Published:** 2023-05-01

**Authors:** Craig Peter Coorey, Luke D. Knibbs, James Otton

**Affiliations:** 1 School of Medicine, Western Sydney University, Campbelltown, NSW 2560, Australia; 2 Royal North Shore Hospital, St Leonards, Sydney, NSW 2065, Australia; 3 Faculty of Medicine and Health, School of Public Health, The University of Sydney, Camperdown, Sydney, NSW 2050, Australia; 4 Public Health Research Analytics and Methods for Evidence, Public Health Unit, Sydney Local Health District, Camperdown, Sydney, NSW 2050, Australia; 5 Department of Cardiology, Liverpool Hospital, Liverpool, NSW 2170, Australia; 6 Faculty of Medicine, South Western Sydney Clinical School, UNSW, Sydney, NSW 2170, Australia

**Keywords:** socioeconomic status, coronary artery calcium, arterial age, coronary artery disease, atherosclerosis

## Abstract

Background: The coronary calcium score is a non-invasive biomarker of coronary artery disease. The concept of “arterial age” transforms the coronary calcium score to an expected age based on the degree of coronary atherosclerosis. This study aimed to investigate the relationship of socioeconomic status with the burden of coronary artery disease within Sydney, Australia. Methods: This was an ecological study at the postcode level of patients aged 45 and above who had completed a CT coronary calcium scan within New South Wales (NSW), Australia from January 2012 to December 2020. Arterial age difference was calculated as arterial age minus chronological age. Socioeconomic data was obtained for median income, Index of Relative Socio-economic Advantage and Disadvantage (IRSAD) score and median property price. Linear regression was used for analysis. Results: There were 17,102 patients across 325 postcodes within NSW, comprising 9129 males with a median arterial age difference of 7 years and 7972 females with -9 years. Income, IRSAD score and property price each had an inverse relationship with arterial age difference (*p*-values < 0.05). Conclusions: Income, socioeconomic status and local property prices are significantly correlated with premature coronary aging. Healthcare resource allocation and prevention should target the inequalities identified to reduce the burden of coronary artery disease.

## 1. Introduction

**Figure 1:**
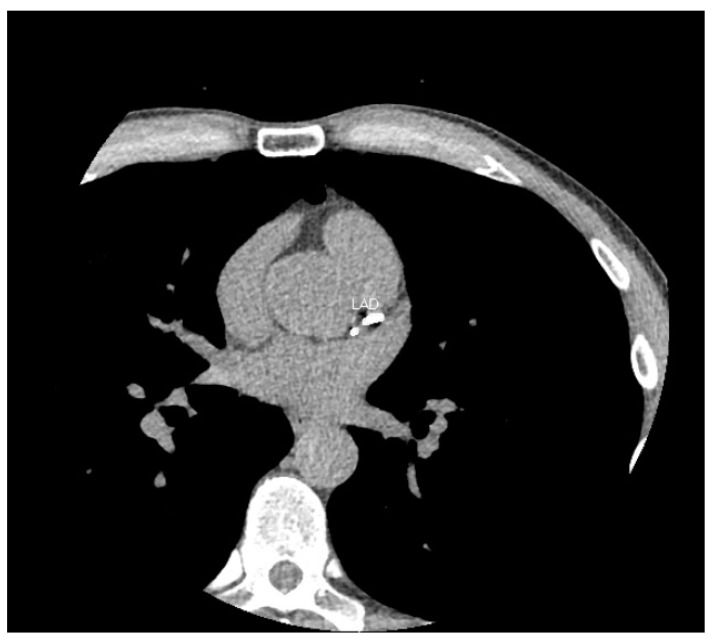
CT calcium score scan. Here is an axial image from a CT calcium score scan of a 51-year-old male. At his age, the majority of males do not exhibit coronary calcification. His total score > 400 places him at greater than the 97th centile for age and gender. The left anterior descending (LAD) artery is labelled.

Ischaemic heart disease secondary to coronary artery disease remains one of the leading causes of death worldwide. The burden of atherosclerotic coronary artery disease is directly correlated to the extent of coronary artery calcium (CAC) [1]. CAC can be measured non-invasively using a non-contrast cardiac CT scan (Figure 1) and has been shown to be an independent predicter of cardiovascular events in multiple studies [2,3]. CAC is often performed as a part of CT coronary angiography studies.

Guidelines from the American Heart Association and American College of Cardiology suggest CAC to be useful in the primary prevention of cardiovascular disease for the risk stratification of individuals whose atherosclerotic cardiovascular disease is borderline (10-year risk of 5 to <7.5%) or intermediate (10-year risk of 7.5 to <20%) [4]. The presence of risk-enhancing factors not captured by the traditional 10-year risk equations also needs to be considered, such as family history of premature cardiovascular disease, primary hypercholesterolaemia, metabolic syndrome, chronic kidney disease and chronic inflammatory conditions. A CAC score of 0 Agatston units (AU) would reclassify an individual’s cardiovascular disease risk to be lower than predicted, whereas a CAC score of 100 AU (or ≥75th percentile for age, sex, race, ethnicity) would reclassify an individual’s cardiovascular disease risk to be higher than predicted and would warrant statin therapy.

In the Australian context, national guidelines have similarly incorporated CAC scores wherein they are mainly used to provide additional cardiovascular disease risk stratification beyond traditional cardiovascular disease risk factors, especially in those patients with moderate risk where there is uncertainty about the intensity of required management or in those patients with low risk where traditional risk factors underestimate the cardiovascular disease risk [5,6]. However, the Australian guidelines use the National Vascular Disease Prevention Alliance (NVDPA) absolute cardiovascular risk algorithm, which predicts the risk of cardiovascular events in a 5-year period, unlike the 10-year period in overseas guidelines.

CAC is highly dependent on age and must be understood in an age-adjusted context [7]. For ease of interpretation, a transformation can be applied to CAC scores to calculate an arterial age. Arterial age is the age-equivalent cardiovascular disease risk for a particular CAC score, derived from the Multi-Ethnic Study of Atherosclerosis cohort study [8]. Arterial age can be compared to biological age as a proxy for the relative excess atherosclerotic disease burden of an individual, adjusted for age. For example, a 45-year-old man with a calcium score of 10 would be deemed to have an arterial age of 56, as this CAC has the same expected coronary artery disease as an average 56-year-old individual. In a cross-sectional study of 241 patients with low and intermediate cardiovascular disease risk and stable angina, CAC scores were found to be less likely to overestimate vascular age compared to other measures such as the Framingham Risk Score and the Systematic Coronary Risk Evaluation [9]. Additionally, arterial age has been shown to be helpful in predicting short-term coronary heart disease events compared to 10-year Framingham risk scores alone [10].

In addition to controlling traditional cardiovascular disease risk factors, there is an increasing interest in the influence of demographic, geographic and socioeconomic factors on cardiovascular disease risk. Multiple national studies have found various socioeconomic factors such as income and level of education to be associated with cardiovascular disease risk [11,12,13,14,15]. These factors, amongst others, can result in a geographical gradient of cardiovascular disease [16]. Identifying such patterns can be used to target public health interventions and reduce the burden of cardiovascular disease.

Australia is a geographically sparse country and has historically had spatial inequality in relation to access to cardiac services across the country [17]. A cross-sectional study of metabolic risk factors for cardiovascular disease in the Illawarra-Shoalhaven region of the NSW, Australia found significant geographical variation, with the highest burden of risk factors along the eastern seaboard [18]. A recent cross-sectional and longitudinal study of 11,035 individuals in Australia found neighbourhood disadvantage to be associated with incidence of self-reported cardiovascular disease and that physical activity was a key mediator of this association [19].

The interplay between socioeconomic inequality, disease prevalence and medical resource availability is complex. Whereas poor cardiovascular outcomes can relate to lack of appropriate treatment or modifying comorbidities, an advantage of the CAC is that it allows the direct visualisation of cardiovascular disease.

This study aimed to explore the relationship of socioeconomic status and geographical location with the burden of coronary artery disease within a localised cohort in Australia. For intuitive clarity, the burden of atherosclerosis was measured using arterial age and its discrepancy with chronological age.

## 2. Materials and Methods

This was a retrospective ecological study of patients who had completed a CT coronary calcium scan at a single private radiology practice (within NSW, Australia) between 1 January 2012 and 7 December 2020. All patients who had a scan between the pre-specified dates were included in this study, irrespective of indication for the scan. The sources of referrals for these scans included cardiologists in private practice and public hospitals. Ethics approval was obtained by the UNSW Human Research Advisory Panel (HC220045). Data collected was coronary calcium score, calcium score percentile rank, gender, age and postcode.

Exclusion criteria were missing calcium scores (except where this data was imputable from other data such as percentile rank), missing postcode, postcode outside of NSW, postcode listed as a “PO Box”, postcode listed as a work address (where known) and those aged 44 or below at time of scan (given that the calculation of arterial age requires age 45 and above).

Arterial age was calculated from the CAC using the following formula: 39.1 + 7.25 × log(calcium score + 1) [8].

Arterial age difference was calculated as arterial age minus chronological age. A positive arterial age difference (>0) suggests that an individual has an excess cardiovascular disease burden relative to their age, whereas a negative arterial age difference (<0) suggests that the cardiovascular disease burden is less than what would be expected at their age.

Data on socioeconomic variables were derived from relevant state and national databases.

Median taxable income in the 2017–2018 financial year at the postcode level was obtained from the Australian Taxation Office [20]. Socioeconomic status at the postcode level was measured by the 2016 Index of Relative Socio-economic Advantage and Disadvantage (IRSAD), extracted from the Australian Bureau of Statistics [21]. This index incorporates 17 variables indicating disadvantage: percentage with low income, no educational attainment, highest level of education is year 11 or lower, highest level of education certificate III/IV, occupation as labourer, occupation machinery operators/drivers, occupation low skill community/personal service worker, occupation low skill sales, families with children who live with jobless parents, long-term disability with need for assistance, unemployment, one parent family with dependent children, paying low rent, separated/divorced, private dwellings without internet connection, private dwellings with no cars and private dwellings requiring extra bedrooms [22]. The index also incorporates 8 variables indicating advantage: percentage with high income, paying high mortgage rates, paying high rent, occupation professional, occupation manager, highest level of education is diploma, studying at university and private dwellings with at least four bedrooms. A high IRSAD score indicates a relative lack of disadvantage and greater advantage.

Median property price at the postcode level was obtained from the NSW Department of Communities and Justice for the period October to December 2020 [23].

Spatial data on boundaries of postcodes was also obtained from the Australian Bureau of Statistics [24].

Regression modelling was used to investigate the association between the arterial age difference and each of the socioeconomic variables (income, IRSAD, property price) at the level of postcode in NSW. A simple linear regression model was creating using a postcode’s median income or IRSAD score or property price as independent variable and a postcode’s median arterial age difference as dependent variable. Given age was already taken into account in the arterial age calculation, it was not further adjusted for in the regression model. For completeness, simple linear regression models were also calculated using the dependent variable median CAC score percentile instead of median age difference. In order to reduce selection bias from postcodes with fewer participants, only those postcodes with at least 15 calcium scores were included in the regression model. Assumptions of linear regression including linearity, homogeneity of variance and normality of residuals were assessed using diagnostic plots.

To explore the geographical variation of arterial age difference, a heatmap was constructed using each postcode’s median arterial age difference, separately for males and females. The heatmap was limited to the Greater Sydney region for visualization. Spatial autocorrelation was calculating using Global Moran’s I statistic, using queen criterion and excluding postcodes without any links.

Statistical analysis was carried out in R version 4.0.2 [25]. Results shown are median (IQR) unless stated otherwise. A significance level of 0.05 was used in all models.

## 3. Results

**Figure 2:**
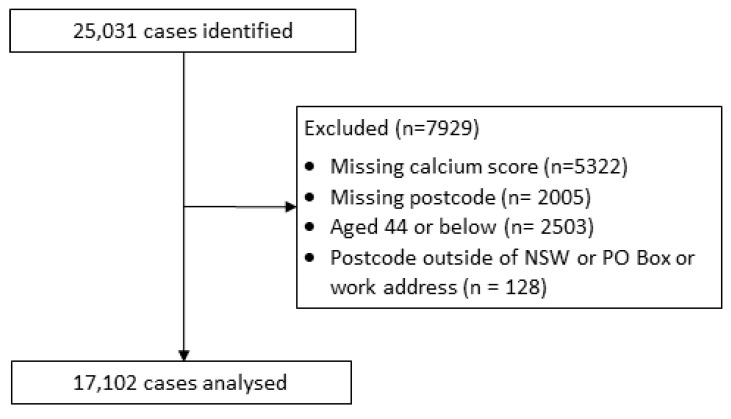
Study population flow diagram.

A total of 25,031 cases were identified. After applying the exclusion criteria, 17,102 cases remained and were included in the subsequent analysis (Figure 2). A total of 325 postcodes within NSW were represented.

Males had a median (IQR) chronological age of 61 years (54 to 68), calcium score of 70 AU (2 to 353), arterial age of 70 years (47 to 82) and arterial age difference of 7 years (−8 to 18) (Table 1), whereas females had a median (IQR) chronological age of 64 years (57 to 70), calcium score of 7 AU (0 to 104), arterial age of 54 years (39 to 73) and arterial age difference of −9 years (−19 to 6).

**Figure 3:**
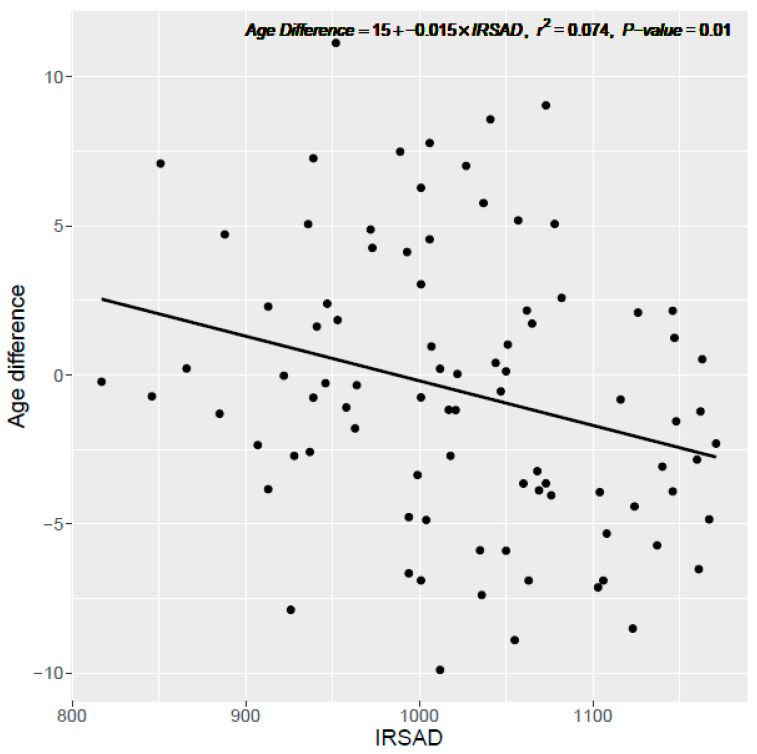
Age difference vs. IRSAD score at the postcode level. Linear regression model of each postcode’s median arterial age difference by Index of Relative Socio-economic Advantage and Disadvantage (IRSAD) score for postcodes within NSW. Age difference calculated as arterial age—chronological age. Only those postcodes with at least 15 patients were included.

For the regression models, 88 postcodes across NSW were included which had at least 15 cases each (total cases 16,179, median 57.5 cases per postcode). Income was found to have an inverse relationship with arterial age difference and to be a significant predictor (Intercept = 6.7, Beta = −1.4 × 10^−4^, Standard error = 4.9 × 10^−5^, *p*-value = 0.004, R^2^ = 0.09) (Table 2). Each AUD 10,000 decrease in income was associated with a 1.4-year increase in arterial age difference. Similarly, inverse relationships were found with property price (Intercept = 1.6, Beta = −2.3 × 10^−6^, Standard error = 1.1 × 10^−6^, *p*-value = 0.04, R^2^ = 0.05) and IRSAD score (Intercept = 14.8, Beta = −0.01, Standard error = 0.006, *p*-value = 0.01, R^2^ = 0.07) (Figure 3). These trends were consistent when using the median CAC score percentile instead of the median age difference (Table S1).

**Figure 4:**
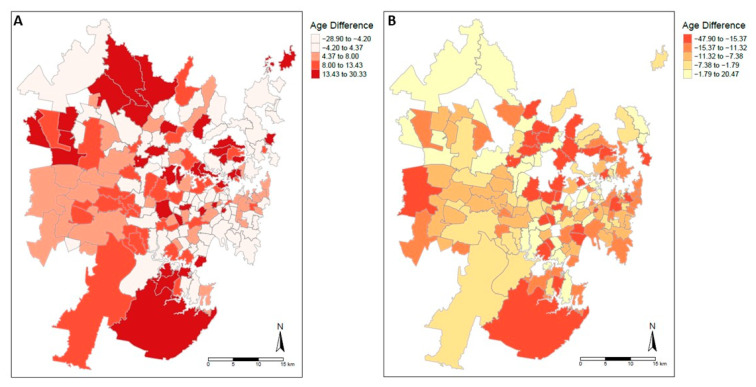
Arterial age difference heatmap. Heatmaps of each postcode’s median arterial age difference across Greater Sydney for males (N = 7670, 177 postcodes, (**A**)) and females (N = 6588, 153 postcodes, (**B**)). Arterial age difference binned into quantiles.

The arterial age difference was mapped for Greater Sydney and heatmaps calculated for males (N = 7670, 177 postcodes) and females (N = 6588, 153 postcodes) (Figure 4). For the heatmap of males, there were areas of large positive age difference in Southern and Western Sydney, with several pockets of large positive age difference in inner Sydney. Most of the postcodes with negative age difference were located in Eastern Sydney, particularly near the coast. For females, there was a similar distribution, except there were several notable postcodes in Southern and Western Sydney which had highly negative age differences.

For the calculation of global spatial autocorrelation, for males, three postcodes were removed, as they did not have any links, and the resulting Moran I statistic was 0.05 with *p*-value 0.14. For females, two postcodes without links were removed and the Moran I statistic was 0.002 with *p*-value 0.44.

## 4. Discussion

This study found that arterial age (when compared to chronological age) was inversely associated with income, local property prices and increasing socioeconomic advantage and decreasing socioeconomic disadvantage in Greater Sydney, NSW, Australia. This finding has important implications for the appropriate targeting of cardiovascular risk factor reduction campaigns, public health responses and resource allocation across heterogenous socioeconomic populations within an urban environment.

In this study, females were slightly older than the males (median age 64 vs. 61 years), yet had markedly lower CAC scores (median CAC 7 vs. 70 AU), resulting in a median negative arterial age difference for females and median positive arterial age difference for males. This is consistent with previous studies wherein males had higher CAC scores than females [7], possibly reflecting the increased cardiovascular risk factors and greater atherosclerotic burden in males.

We found a socioeconomic gradient in arterial age by using a composite socioeconomic score (IRSAD) that included multiple variables covering income, employment, education, disability support and dwelling/vehicle ownership. This score is specific to the Australian context and validated by the Australian Bureau of Statistics [22]. We additionally investigated the relationship of CAC scores with income tax and property price, which were consistent with the relationship demonstrated by the IRSAD score. Additionally, although there was no significant global spatial autocorrelation detected, the heatmaps for the Greater Sydney area demonstrated pockets of highly positive and negative arterial age difference, similar for both males and females.

The finding of a socioeconomic gradient in arterial age is consistent with previous studies which have found similar trends of CAC correlation with socioeconomic status. A cross-sectional study in Sweden of 1067 individuals aged 50 to 64 years old with no history of coronary artery disease found those living in low socioeconomic areas had a higher proportion of non-zero CAC scores compared to those living in high socioeconomic areas (46.3% vs. 36.6%) [26]. When adjusted for age, gender and cardiovascular disease risk factors, this association was not significant, suggesting that socioeconomic status may influence CAC scores via modifying cardiovascular disease risk factors. A subgroup analysis of 528 asymptomatic individuals from the Whitehall II epidemiological cohort study in London used grade of employment as an indicator of socioeconomic status and found the severity of CAC (but not its presence) was inversely associated with grade of employment, even after controlling for lifestyle, biological and psychosocial factors [27].

Identifying the risk factors driving the observed socioeconomic gradient in arterial age may allow for targeted public health interventions and redistribution of healthcare resources aimed at reducing the burden of ischaemic heart disease. Further studies are needed to investigate the impact and interaction of socioeconomic variables and traditional cardiovascular disease risk factors on these observed gradients in NSW, Australia. It would also be important to capture socioeconomic variables not included in the IRSAD such as psychosocial factors and behavioural factors that are known to impact cardiovascular disease [28]. A recent study of 137,408 patients with coronary heart disease treated in general practices in Australia found that patients with lower socioeconomic status were less likely to achieve treatment goals for the secondary prevention of cardiovascular disease despite being more likely to be prescribed medications for secondary prevention [29].

There has been progress in Australia in addressing the socioeconomic inequity regarding cardiovascular disease, with a retrospective study of ischaemic heart disease mortality in Australia over the period 1979 to 2006 reporting a narrowing of the difference in mortality rates between females from low and high socioeconomic status, but not for males [30]. Given we have demonstrated a socioeconomic gradient over a postcode level, public health interventions aimed at narrowing the socioeconomic gradient (with the aim of reducing the ischaemic heart disease burden) could be targeted to a postcode level as well. A retrospective cohort study of 178,812 individuals with a median follow-up of 10 years in the Republic of Korea showed that an upward shift in socioeconomic status (as defined by income) during the follow-up period was associated with reduced cardiovascular mortality, even after adjusting for a range of traditional cardiovascular disease risk factors [31].

Previous studies have shown geographical inequality in traditional cardiovascular disease risk factors, particularly in Australia. For diabetes mellitus, a prior study of 114,755 adults in Sydney found the odds of having diabetes mellitus varied geographically by 42% [32]. A systematic review of 20 studies demonstrated an increased ischaemic heart disease burden in regional and remote areas compared to major cities in Australia [33]. Discrepancies between the geographical distribution of CAC and traditional cardiovascular disease risk factors may be driven by non-traditional cardiovascular disease risk factors, including socioeconomic factors already mentioned as well as other emerging cardiovascular disease risk factors like air pollution.

A number of studies have found associations between air pollution exposure and CAC scores, but the results have not been consistent. An analysis of baseline data of 4494 individuals from the German Heinz Nixdorf Recall Study found that living near a major road was associated with higher CAC scores, even after controlling for particulate matter < 2.5 µm (PM2.5) and cardiovascular disease risk factors [34]. However, in this study the association between PM2.5 and CAC did not reach statistical significance. In a prospective longitudinal cohort study of 6795 individuals in the United States of America, increases in long-term exposure to both PM2.5 and nitrous oxides were associated with increasing CAC score progression [35]. A recent cohort study in Sweden of 5070 individuals aged 50 to 65 years old found higher CAC scores were associated with traffic-related air pollutants but not long-term exposure to ambient air pollution (PM2.5) [36]. In the Australian context, a study of 666 asymptomatic adults across three states in Australia found that long-term exposure to air pollution such as PM2.5 and nitrogen dioxide (NO_2_) was associated with higher CAC scores, independent of other risk factors [37]. It would be interesting to measure the long-term air pollution exposure for the participants in our study, such as estimation of PM2.5 exposure with a land-use regression model, to determine whether the geospatial distribution of CAC scores is associated with long-term air pollution exposure [38].

An additional factor that could influence the measured geographical distribution of cardiovascular disease is the under-diagnosis of cardiovascular disease in some postcodes. Although CAC scores are agnostic to postcode, there may be variation in which patients receive cardiac CT scans depending on socioeconomic factors, availability of general practitioners and cardiologists and location/accessibility of radiology practices. A cross-sectional study in England of almost 10 million patients found there to be significant geospatial variability in the observed and expected prevalence of cardiovascular diseases, possibly driven by inequity in the supply of general practitioners [39]. Future ecological studies could investigate for this influence by adjusting for factors such as the number of general practitioners and cardiologists in each postcode.

A strength of this study is the large sample of approximately 17,000 individuals from >300 postal areas across NSW. This has allowed analysis of the coronary artery disease burden over a wide range of socioeconomic backgrounds and geographical locations. Analysing data at the postcode level allowed the use of a validated measure of socioeconomic status (IRSAD score).

Given this was an ecological study, we do not have individual-level data on traditional cardiovascular disease risk factors and are unable to adjust for these in the analysis. Postcode data was taken at the time of the scan and may not represent the patient’s long-term address if they have moved recently or if they have listed their work address instead of home address (where possible, work addresses were excluded). Given the duration of study recruitment, the socioeconomic data could only be extracted at a single timepoint, and it is possible that socioeconomic status could have changed during the study period.

Although it has been demonstrated that males and females have different arterial age scores, we are unable to adjust for gender in our regression models given this study was at a postcode level and the outcomes (IRSAD, income, property price) were not available split by gender at the postcode level. However, we do not expect this would have had a large impact on the trends elucidated, given arterial age would have been systematically over- and underestimated to the same extent for all of the postcodes.

A limitation of this ecological study is that we cannot state the causation of the discovered premature coronary disease. On the other hand, a strength of the use of calcium score data is that they provide an objective measure of the underlying pathology of atherosclerosis and are not biased by the treatment effects or healthcare resource availability that may affect other cardiovascular outcome measures.

## 5. Conclusions

We have shown that in NSW, Australia, there is an inverse association between socioeconomic status and cardiovascular disease risk. Premature coronary atherosclerosis is significantly correlated with reducing income and local property prices. This may have implications for healthcare resource allocation and preventative healthcare strategies.

## Figures and Tables

**Table 1:** Summary data split by gender. Results shown are median with interquartile range (IQR). Note that one patient had “other” gender recorded.

	Total	Males	Females
N	17,102	9129	7972
Chronological age, *median (IQR)*	62 (55 to 69)	61 (54 to 68)	64 (57 to 70)
Calcium score, *median (IQR)*	31.0 (0.0 to 223.0)	70.0 (2.0 to 353.0)	7.0 (0.0 to 104.2)
Arterial Age, *median (IQR)*	64.23 (39.10 to 78.33)	70.00 (47.06 to 81.65)	54.18 (39.10 to 72.86)
Arterial age difference, *median (IQR)*	−0.36 (−14.76 to 13.11)	6.79 (−7.91 to 17.78)	−8.90 (−18.90 to 5.52)

**Table 2:** Regression models for age difference at the postcode level. Univariate regression models of each postcode’s median socioeconomic variable (IRSAD, income, property price) as predictor and each postcode’s median age difference as outcome. Age difference calculated as arterial age—chronological age. IRSAD: Index of Relative Socio-economic Advantage and Disadvantage.

	Intercept	Beta	Standard Error	*p*-Value	R-Squared Value
IRSAD Score	14.8	−0.015	0.006	0.01	0.07
Income	6.7	−1.4 × 10^−4^	4.9 × 10^−5^	0.004	0.09
Property Price	1.6	−2.3 × 10^−6^	1.1 × 10^−6^	0.04	0.05

## Data Availability

The datasets used and/or analysed during the current study are available from the corresponding author on reasonable request.

## Supplementary Materials

The following supporting information can be downloaded at: https://www.mdpi.com/article/10.3390/ijerph20095699/s1, Table S1: Regression models for CAC percentile at the postcode level.

## Abbreviations

CAC	Coronary artery calcium
IRSAD	Index of Relative Socio-economic Advantage and Disadvantage
IQR	Interquartile range
NSW	New South Wales

## References

[1] Wexler L., Brundage B., Crouse J., Detrano R., Fuster V., Maddahi J., Rumberger J., Stanford W., White R., Taubert K. (1996). Coronary artery calcification: Pathophysiology, epidemiology, imaging methods, and clinical implications: A statement for health professionals from the American Heart Association. Circulation.

[2] Pletcher M.J., Tice J.A., Pignone M., Browner W.S. (2004). Using the coronary artery calcium score to predict coronary heart disease events: A systematic review and meta-analysis. Arch. Intern. Med..

[3] McEvoy J.W., Blaha M.J., DeFilippis A.P., Budoff M.J., Nasir K., Blumenthal R.S., Jones S.R. (2010). Coronary artery calcium progression: An important clinical measurement? A review of published reports. J. Am. Coll. Cardiol..

[4] Lloyd-Jones D.M., Braun L.T., Ndumele C.E., Smith S.C., Sperling L.S., Virani S.S., Blumenthal R.S. (2019). Use of risk assessment tools to guide decision-making in the primary prevention of atherosclerotic cardiovascular disease: A special report from the American Heart Association and American College of Cardiology. Circulation.

[5] Hamilton-Craig C.R., Chow C.K., Younger J.F., Jelinek V.M., Chan J., Liew G.Y. (2017). Cardiac Society of Australia and New Zealand position statement executive summary: Coronary artery calcium scoring. Med. J. Aust..

[6] Jennings G.L., Audehm R., Bishop W., Chow C.K., Liaw S.T., Liew D., Linton S.M. (2021). National Heart Foundation of Australia: Position statement on coronary artery calcium scoring for the primary prevention of cardiovascular disease in Australia. Med. J. Aust..

[7] McClelland R.L., Chung H., Detrano R., Post W., Kronmal R.A. (2006). Distribution of coronary artery calcium by race, gender, and age: Results from the Multi-Ethnic Study of Atherosclerosis (MESA). Circulation.

[8] McClelland R.L., Nasir K., Budoff M., Blumenthal R.S., Kronmal R.A. (2009). Arterial age as a function of coronary artery calcium (from the Multi-Ethnic Study of Atherosclerosis [MESA]). Am. J. Cardiol..

[9] Vecsey-Nagy M., Szilveszter B., Kolossváry M., Boussoussou M., Vattay B., Merkely B., Maurovich-Horvat P., Radovits T., Nemcsik J. (2022). Correlation between coronary artery calcium-and different cardiovascular risk score-based methods for the estimation of vascular age in caucasian patients. J. Clin. Med..

[10] Villines T.C., Taylor A.J. (2012). Multi-ethnic study of atherosclerosis arterial age versus framingham 10-year or lifetime cardiovascular risk. Am. J. Cardiol..

[11] Lynch J.W., Kaplan G.A., Cohen R.D., Tuomilehto J., Salonen J.T. (1996). Do cardiovascular risk factors explain the relation between socioeconomic status, risk of all-cause mortality, cardiovascular mortality, and acute myocardial infarction?. Am. J. Epidemiol..

[12] Bobak M., Hertzman C., Skodova Z., Marmot M. (1999). Socioeconomic status and cardiovascular risk factors in the Czech Republic. Int. J. Epidemiol..

[13] Metcalf P.A., Scragg R.R., Schaaf D., Dyall L., Black P.N., Jackson R.T. (2008). Comparison of different markers of socioeconomic status with cardiovascular disease and diabetes risk factors in the Diabetes, Heart and Health Survey. N. Z. Med. J..

[14] Kestilä P., Magnussen C.G., Viikari J.S., Kähönen M., Hutri-Kähönen N., Taittonen L., Jula A., Loo B.M., Pietikäinen M., Jokinen E. (2012). Socioeconomic status, cardiovascular risk factors, and subclinical atherosclerosis in young adults: The cardiovascular risk in Young Finns Study. Arterioscler. Thromb. Vasc. Biol..

[15] Jaffiol C., Thomas F., Bean K., Jégo B., Danchin N. (2013). Impact of socioeconomic status on diabetes and cardiovascular risk factors: Results of a large French survey. Diabetes Metab..

[16] Casper M., Kramer M.R., Quick H., Schieb L.J., Vaughan A.S., Greer S. (2016). Changes in the geographic patterns of heart disease mortality in the United States: 1973 to 2010. Circulation.

[17] Clark R.A., Coffee N., Turner D., Eckert K.A., Van Gaans D., Wilkinson D., Stewart S., Tonkin A.M. (2012). Application of geographic modeling techniques to quantify spatial access to health services before and after an acute cardiac event: The Cardiac Accessibility and Remoteness Index for Australia (ARIA) project. Circulation.

[18] Toms R., Mayne D.J., Feng X., Bonney A. (2019). Geographic variation in cardiometabolic risk distribution: A cross-sectional study of 256,525 adult residents in the Illawarra-Shoalhaven region of the NSW, Australia. PLoS ONE.

[19] Saghapour T., Giles-Corti B., Rachele J., Turrell G. (2021). A cross-sectional and longitudinal study of neighbourhood disadvantage and cardiovascular disease and the mediating role of physical activity. Prev. Med..

[20] Australian Taxation Office Taxation Statistics 2017–2018. https://www.ato.gov.au/.

[21] Australian Bureau of Statistics (2016). Socio-Economic Indexes for Areas (SEIFA). https://www.ausstats.abs.gov.au.

[22] Australian Bureau of Statistics (2016). Technical Paper: Socio-Economic Indexes for Areas (SEIFA). https://www.intelia.com.au/wp-content/uploads/2020/09/SEIFA-2016-Technical-Paper.pdf.

[23] NSW Department of Communities and Justice Report Number 135 December Quarter 2020 Sales Tablets. https://www.facs.nsw.gov.au/.

[24] Australian Bureau of Statistics Australian Statistical Geography Standard (ASGS). https://www.abs.gov.au/.

[25] R Core Team (2022). R: A Language and Environment for Statistical Computing.

[26] Djekic D., Angerås O., Lappas G., Fagman E., Fagerberg B., Bergström G., Rosengren A. (2018). Impact of socioeconomic status on coronary artery calcification. Eur. J. Prev. Cardiol..

[27] Steptoe A., Hamer M., O’Donnell K., Venuraju S., Marmot M.G., Lahiri A. (2010). Socioeconomic status and subclinical coronary disease in the Whitehall II epidemiological study. PLoS ONE.

[28] Clark A.M., DesMeules M., Luo W., Duncan A.S., Wielgosz A. (2009). Socioeconomic status and cardiovascular disease: Risks and implications for care. Nat. Rev. Cardiol..

[29] Mnatzaganian G., Lee C.M.Y., Robinson S., Sitas F., Chow C.K., Woodward M., Huxley R.R. (2021). Socioeconomic disparities in the management of coronary heart disease in 438 general practices in Australia. Eur. J. Prev. Cardiol..

[30] Page A., Lane A., Taylor R., Dobson A. (2012). Trends in socioeconomic inequalities in mortality from ischaemic heart disease and stroke in Australia, 1979–2006. Eur. J. Prev. Cardiol..

[31] Sung J., Song Y.M., Hong K.P. (2020). Relationship between the shift of socioeconomic status and cardiovascular mortality. Eur. J. Prev. Cardiol..

[32] Astell-Burt T., Feng X., Kolt G.S., McLean M., Maberly G. (2014). Understanding geographical inequities in diabetes: Multilevel evidence from 114,755 adults in Sydney, Australia. Diabetes Res. Clin. Pract..

[33] Alston L., Allender S., Peterson K., Jacobs J., Nichols M. (2017). Rural inequalities in the Australian burden of ischaemic heart disease: A systematic review. Heart Lung Circ..

[34] Hoffmann B., Moebus S., Mohlenkamp S., Stang A., Lehmann N., Dragano N., Schmermund A., Memmesheimer M., Mann K., Erbel R. (2007). Residential exposure to traffic is associated with coronary atherosclerosis. Circulation.

[35] Kaufman J.D., Adar S.D., Barr R.G., Budoff M., Burke G.L., Curl C.L., Daviglus M.L., Roux A.V.D., Gassett A.J., Jacobs D.R. (2016). Association between air pollution and coronary artery calcification within six metropolitan areas in the USA (the Multi-Ethnic Study of Atherosclerosis and Air Pollution): A longitudinal cohort study. Lancet.

[36] Edlund K.K., Sallsten G., Molnár P., Andersson E.M., Ögren M., Segersson D., Fagman E., Fagerberg B., Barregard L., Bergström G. (2022). Long-term exposure to air pollution, coronary artery calcification, and carotid artery plaques in the population-based Swedish SCAPIS Gothenburg cohort. Environ Res..

[37] Huynh Q., Marwick T.H., Venkataraman P., Knibbs L.D., Johnston F.H., Negishi K. (2021). Long-term exposure to ambient air pollution is associated with coronary artery calcification among asymptomatic adults. Eur. Heart J. Cardiovasc. Imaging.

[38] Knibbs L.D., Van Donkelaar A., Martin R.V., Bechle M.J., Brauer M., Cohen D.D., Cowie C.T., Dirgawati M., Guo Y., Hanigan I.C. (2018). Satellite-based land-use regression for continental-scale long-term ambient PM2. 5 exposure assessment in Australia. Environ. Sci. Technol..

[39] Soljak M., Samarasundera E., Indulkar T., Walford H., Majeed A. (2011). Variations in cardiovascular disease under-diagnosis in England: National cross-sectional spatial analysis. BMC Cardiovasc. Disord..

