# Supplementary material for: Social, Geographical and Income Inequality as Demonstrated by the Coronary Calcium Score: An Ecological Study in Sydney, Australia
Source: Int J Environ Res Public Health. 2023 May 1;20(9):5699. doi: 10.3390/ijerph20095699 (PMC10178035; doi:10.3390/ijerph20095699)
Supplement: Supplementary file 1 [file ijerph-20-05699-s001.zip › ijerph-2309707-supplementary.pdf]

Supplementary data

Table S1. Regression models for CAC percentile at the postcode level. Univariate regression models of each postcode's median socioeconomic variable (IRSAD, income, property price) as predictor and each postcode's median coronary artery calcium (CAC) score percentile as outcome. IRSAD: Index of Relative Socio-economic Advantage and Disadvantage.

	Intercept	Beta	Standard Error	P-value	R-Squared Value
IRSAD Score	90.2	−0.020	0.008	0.01	0.07
Income	79.4	-1.9×10^{-4}	6.5×10^{-5}	0.004	0.09
Property Price	74.0	-4.2×10^{-6}	1.4×10^{-6}	0.004	0.1
